# HPB News

**DOI:** 10.1155/1989/29406

**Published:** 1989


*HPB Surgery* 1989, Vol. 1, pp. 259–262
Reprints available directly from the publisher
Photocopying permitted by license only

© 1989 Harwood Academic Publishers GmbH
Printed in the United Kingdom

### HPB NEWS

### 3rd WORLD CONGRESS ON HEPATO-PANCREATO-BILIARY SURGERY, 3–8 JUNE, 1990, LONDON, UK

### *Scientific programme*

The congress will take place in Kensington Town Hall, London, UK. Papers of high quality from all over the world will be presented and discussed. *Papers are encouraged from developing countries, young investigators and those with work in progress.* Panels of experts will tackle some of the most controversial areas in HPB surgery. Videos and films may be submitted for presentation. Contributions are invited in areas of HPB surgical or non-surgical technique and also in all other areas of HPB research. *Poster presentations will form a major part of the meeting* and selected posters will be discussed in open sessions. Several world leaders in HPB surgery or medicine will give key note addresses. Among these are Nancy Ascher — liver transplantation, Henry Bismuth — portal hypertension, John Cameron — cholangiocarcinoma, David Carter — pancreatitis, Arthur Li — primary liver tumours, Miles Little — HPB trauma, Dame Sheila Sherlock — hepatitis, Michael Trede — carcinoma of the pancreas.

The language of the meeting will be English. Abstracts will be published in a supplement to HPB Surgery.

### *Social programme*

There will be a welcome reception during the evening of Sunday, June 3 at Kensington Town Hall. The congress banquet will be held at Whitbread Brewery, a very old and historical building in the heart of the city of London. *An evening will be left free for participants from each country to organize meetings of their own chapters.* The congress staff will be happy to help organize these events.

Everyone who wishes to participate can write to the HPB Congress Secretariat for copies of the call for abstracts and accompanying forms. The address to the secretariat is: HPB Congress Secretariat, Congress House, 65 West Drive, Sutton, Surrey, SM2 7NB, United Kingdom.

Contact Miss Caroline Roney, telephone number international 44–1–6610877, fax international 44–1–6619036.

The organizing committee of the meeting consists of the following members: R.C.N. Williamson (Chairman), I.S. Benjamin, K.E.F. Hobbs, M.C.A. Puntis, B.J. Rowlands and J.M. Thompson.

A large scientific committee organizes the scientific programme: C. Broelsch (Chairman), USA; I.S. Benjamin, UK; P.J. Fabri, USA; D. Franco, France; J. Ham, Australia; K.E.F. Hobbs, UK; B. Jeppsson, Sweden; B. Kremer, FRG; P.F. Lam, Hong Kong; H. Obertop, The Netherlands; M.C.A. Puntis, UK; J. Scheele, FRG; P.H. Sugarbaker, USA; O. Terpstra, The Netherlands; T. Tobe, Japan.

### THE WORLD ASSOCIATION OF HPB SURGERY

Executive council members:

Martin A. Adson, M.D. — President
Mayo Clinic
200 First Street S.W.
Rochester, Minnesota 55905
USA
tel. 1–507–2842691
fax. 1–507–2844223

Professor Stig Bengmark
Department of Surgery
Lund University
S–221 85 Lund
SWEDEN
tel. 46–46–171160
fax. 46–46–172335

Christoph Broelsch, M.D.
Department of Surgery
University of Chicago
5841 South Maryland Avenue
Chicago, Illinois 60637
USA
tel. 1–312–7026424
fax. 1–312–7021225

Bernard Langer, M.D.
Department of Surgery
Toronto General Hospital
200 Elizabeth Street
Toronto, Ontario M5G 1L7
CANADA
tel. 1–416–5953554
fax. 1–416–5959486

Arthur K.C. Li, M.D.
Department of Surgery

Prince of Wales Hospital
Shatin, N.T.
HONG KONG
tel. 852–0–6362624
fax. 852–0–6370979

J.M. Little, M.D.
Department of Surgery
Westmead Hospital
Westmead, NSW 2145
AUSTRALIA
tel. 61–2–6336025
fax. 61–2–6334984

Henry A. Pitt, M.D.
Department of Surgery
The Johns Hopkins Hospital
Blalock 688
Baltimore, Maryland 21205
USA
tel. 1–301–9557794
fax. 1–301–9550834

M.N. Van der Heyde, M.D.
Department of Surgery
Academisch Ziekenhuis
Meibergdreef 9
NL–1105 AZ Amsterdam Zuidoost
THE NETHERLANDS
tel. 31–20–5662166
fax. 31–20–5664440

Robin Williamson, M.D.
Department of Surgery
Hammersmith Hospital
Du Cane Road
London W12 OHS
GREAT BRITAIN
tel. 44–1–7403210
fax. 44–1–7403179

Do communicate with your council member.

### 1992 HPB MEETING — HONG KONG

The 4th world congress on hepato-pancreato-biliary surgery will be arranged Sunday May 31, 1990 to Saturday June 6 in Hong Kong. The meeting will take place at the newly constructed Hong Kong Convention and Exhibition Center. A group of members representing the whole surgical committee in Hong Kong will be responsible for the local arrangements. It will be chaired by Professor Arthur Li.

### FUTURE HPB CONFERENCES

The general committee of the HPB World Association agreed in principal that a 1994 meeting should be arranged for the American continent. Two invitations have been received, one from Toronto (Bernard Langer) and another from Boston (Blake Cady). An invitation has also been received for 1996 from Greece (Basil Kekis).

